# Phylogeny Predicts the Quantity of Antimalarial Alkaloids within the Iconic Yellow *Cinchona* Bark (Rubiaceae: *Cinchona calisaya*)

**DOI:** 10.3389/fpls.2017.00391

**Published:** 2017-03-22

**Authors:** Carla Maldonado, Christopher J. Barnes, Claus Cornett, Else Holmfred, Steen H. Hansen, Claes Persson, Alexandre Antonelli, Nina Rønsted

**Affiliations:** ^1^Natural History Museum of Denmark, University of CopenhagenCopenhagen, Denmark; ^2^Herbario Nacional de Bolivia, Universidad Mayor de San AndresLa Paz, Bolivia; ^3^Analytical Biosciences, Department of Pharmacy, Faculty of Health and Medical Sciences, University of CopenhagenCopenhagen, Denmark; ^4^Department of Biological and Environmental Sciences, University of GothenburgGothenburg, Sweden; ^5^Gothenburg Global Biodiversity CentreGothenburg, Sweden; ^6^Gothenburg Botanical GardenGothenburg, Sweden

**Keywords:** alkaloids, Bolivia, *Cinchona calisaya*, climate, plant chemical defense, phylogeny, plant–climate interactions, quinine

## Abstract

Considerable inter- and intraspecific variation with respect to the quantity and composition of plant natural products exists. The processes that drive this variation remain largely unknown. Understanding which factors determine chemical diversity has the potential to shed light on plant defenses against herbivores and diseases and accelerate drug discovery. For centuries, *Cinchona* alkaloids were the primary treatment of malaria. Using *Cinchona calisaya* as a model, we generated genetic profiles of leaf samples from four plastid (trnL-F, matK, rps16, and ndhF) and one nuclear (ITS) DNA regions from twenty-two *C. calisaya* stands sampled in the Yungas region of Bolivia. Climatic and soil parameters were characterized and bark samples were analyzed for content of the four major alkaloids using HPLC-UV to explore the utility of evolutionary history (phylogeny) in determining variation within species of these compounds under natural conditions. A significant phylogenetic signal was found for the content of two out of four major *Cinchona* alkaloids (quinine and cinchonidine) and their total content. Climatic parameters, primarily driven by changing altitude, predicted 20.2% of the overall alkaloid variation, and geographical separation accounted for a further 9.7%. A clade of high alkaloid producing trees was identified that spanned a narrow range of altitudes, from 1,100 to 1,350 m. However, climate expressed by altitude was not a significant driver when accounting for phylogeny, suggesting that the chemical diversity is primarily driven by phylogeny. Comparisons of the relative effects of both environmental and genetic variability in determining plant chemical diversity have scarcely been performed at the genotypic level. In this study we demonstrate there is an essential need to do so if the extensive genotypic variation in plant biochemistry is to be fully understood.

## Introduction

**Figure 1:**
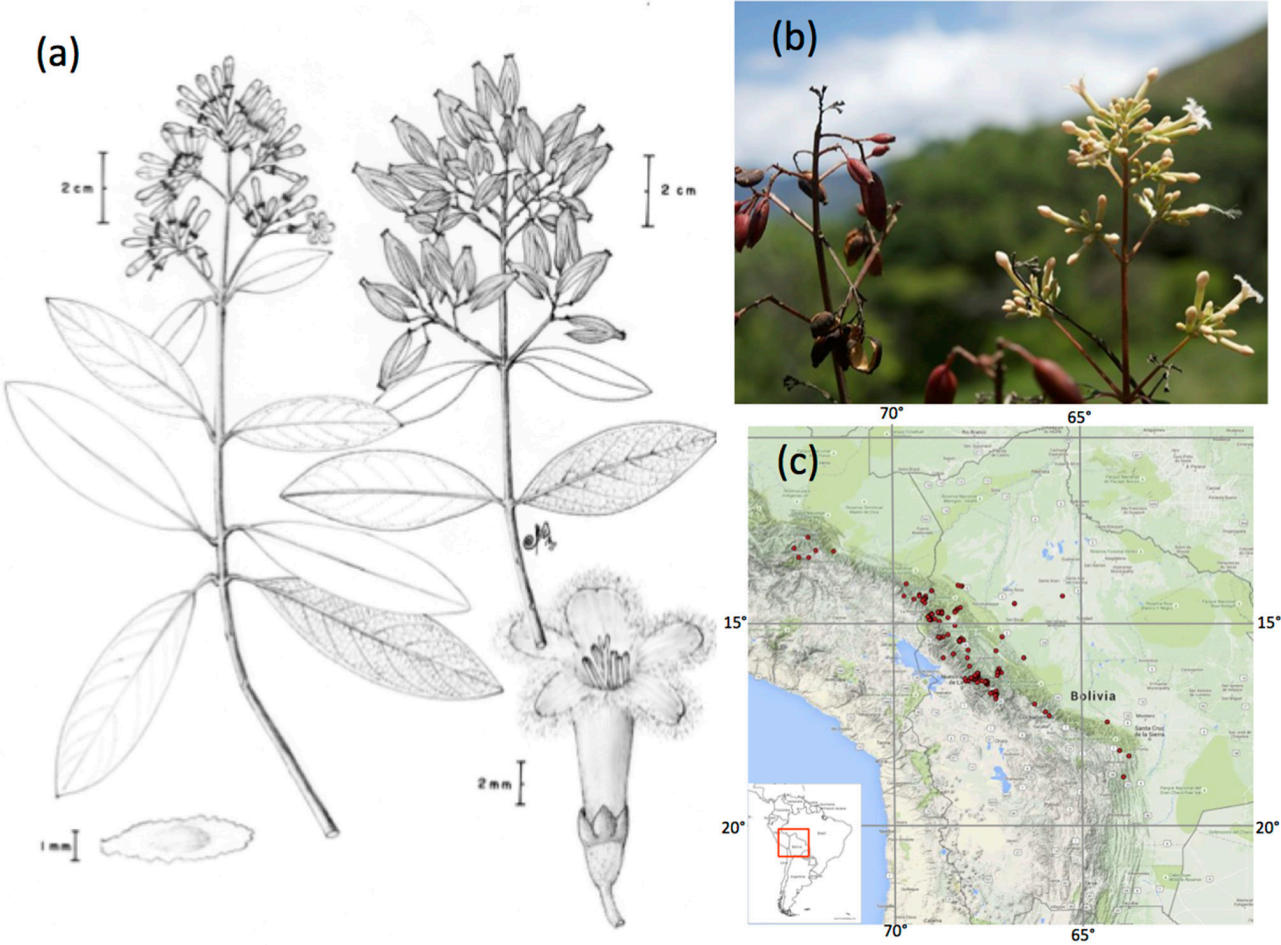
**The yellow Cinchona bark, ***Cinchona calisaya*** (Rubiaceae)**. **(A)** Fertile details of a branch depicting flowers, fruits, and seeds. Drawing from Maldonado 4216 by Carlos Maldonado, **(B)** Photo of one specimen collected in Bolivia (Maldonado 4242) Photo: Alexandre Antonelli, **(C)** Distribution of *C. calisaya* along the eastern Andes of Bolivia and Peru (inset: Position in South America).

Bark from *Cinchona* trees (*Cinchon*a L., Rubiaceae) of the Andean mountain forests produce quinine alkaloids, which were the only effective treatment of malaria for more than four centuries (Honigsbaum, 2001; Kaufman and Ruveda, 2005). The medicinal value of *Cinchona* bark was first discovered in Loxa (now Loja, Ecuador) in the seventeenth century by Jesuit monks, and soon exports of different varieties of *Cinchona pubescens* Vahl (red bark) from South America to Europe were reaching half a million kilograms bark per year (Roersch van der Hoogte and Pieters, 2015). Import could not meet demand, and a quest began for the most productive source of *Cinchona* trees to establish plantations by the British, Dutch, and French empires. The Bolivian *Cinchona calisaya* Wedd. (yellow bark, Figure 1) proved to be the most productive species known to date (Greenwood, 1992; Nair, 2010).

**Figure 2:**
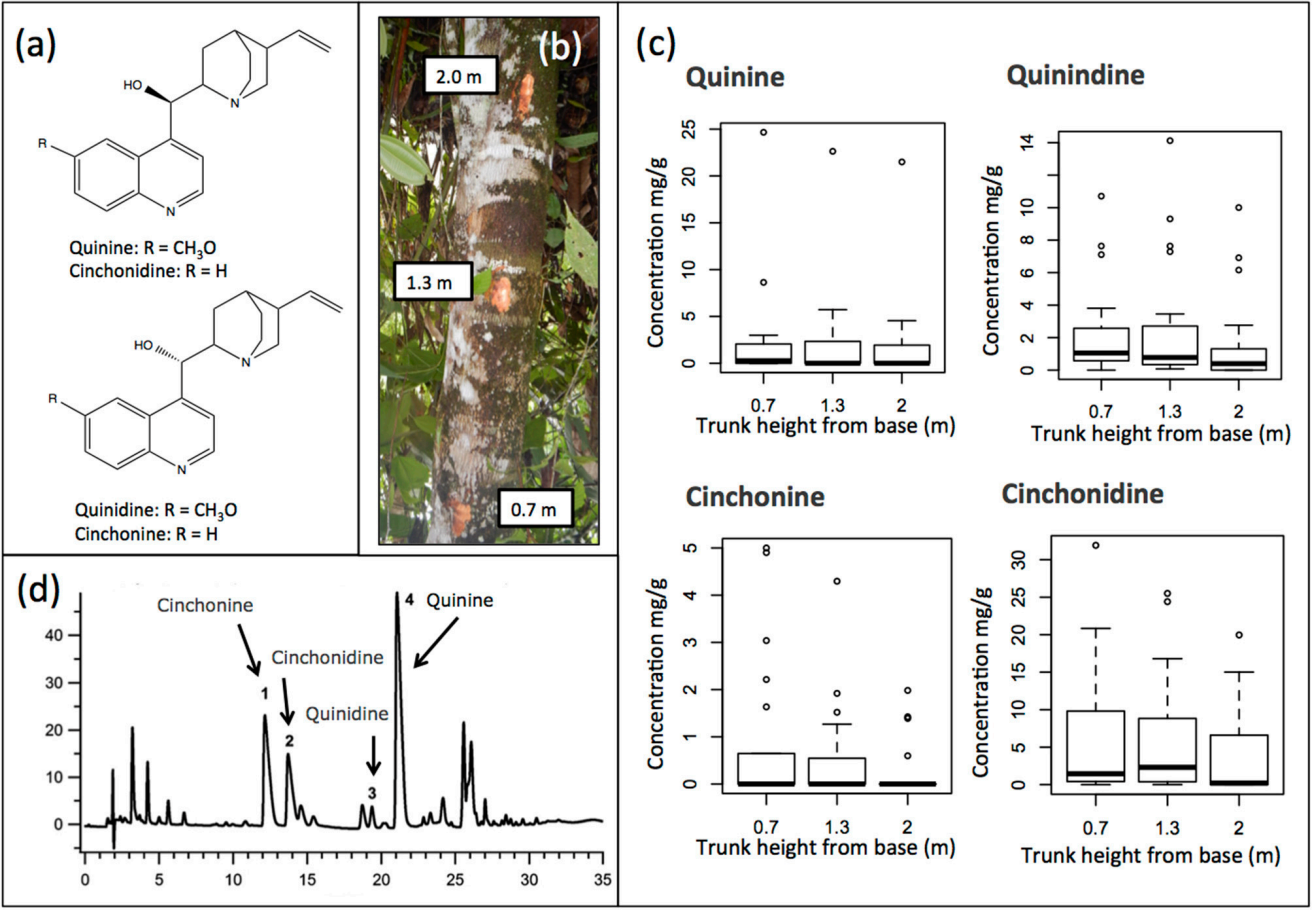
**(a)** Structure of the four major *Cinchona* alkaloids: Quinine, quinidine, cinchonine, and cinchonidine, **(b)** Collection of bark at three different heights of the trunk (0.7, 1.3, and 2.0 m), **(c)** Example of chromatogram of an extract of *Cinchona calisaya* showing the peaks of the four major *Cinchona* alkaloids: (1) cinchonine, (2) cinchonidine, (3) quinidine, and (4) quinine, **(d)** Box plots showing the quantity of the four alkaloids at three different heights across all the samples. No significant differences were detected.

*C. calisaya* is one of 23 species of trees in the genus *Cinchona* described to date, which produce varying amounts of alkaloids. The four major *Cinchona* alkaloids (quinine, quinidine, cinchonine, and cinchonidine) (Figure 2a) all possess antimalarial activity but have different pharmacological profiles (Taggart et al., 1948; Hill, 1963; Bruce-Chwatt, 1990). Since the first isolation of quinine in 1820 over 30 minor and less studied *Cinchona* alkaloids have been described from the genus (Kacprzak, 2013). Bark and roots are the main source of *Cinchona* alkaloids, whereas cinchophyllines are reported from leaves (Aerts et al., 1991). The site of production of the alkaloids has not been established. In addition, *Cinchona* type alkaloids have also been found in the related genera *Ladenbergia* Klotzsch and *Remijia* DC (Okunade et al., 2001; Ruiz-Mesia et al., 2005; Cosenza et al., 2013).

*Cinchona* bark and its alkaloids remained the most efficient treatment of malaria until the 1940s when chloroquine and other synthetic antimalarial compounds were developed (Newman et al., 2000; Kaufman and Ruveda, 2005). With the development of resistant malaria strains (Bruce-Chwatt, 1990) the quest for new antimalarial compounds is continuing, and the discovery of artemisinin from a Chinese herbal medicine based on *Artemisia annua* L. (Tu, 2011), was rewarded with the Nobel prize in medicine in 2015.

Quinine content does not only vary among species (Nair, 2010), but also among populations from different sites, complicating the identification of the most productive *Cinchona* barks (Townley, 1922; Holland, 1932). Natural variation in quinine content remains unexplained, as few studies have been conducted in natural habitats until now (Rusby, 1931; Hodge, 1946; Bruce-Chwatt, 1990).

Alkaloids are a large and varied family of nitrogen-containing natural products occurring widespread across several lineages of vascular plant species with a high degree of specificity of subtypes to plant lineages (Zulak et al., 2006). They are not essential for primary metabolism, but play a number of specialized roles within the plants. Through selective up or down regulation, alkaloids can vary from complete absence to very high concentrations among individuals of the same species (Moore et al., 2014). Alkaloid production is one of the primary mechanisms of plants response to environmental changes (Theis and Lerdau, 2003; Ramakrishna and Ravishankar, 2011), and as with other plant chemical defenses, have likely developed over different evolutionary timescales (Becerra et al., 2009). Evolutionary approaches have been successfully implemented in predicting plant phytochemical composition, accelerating the discovery, and exploration of plant-based medicines (Bohlin et al., 2010; Zhu et al., 2011; Rønsted et al., 2012). However, other studies have found inconsistency of specialized metabolite profiles at various taxonomic levels (e.g., Wink, 2003; Wink and Mohamed, 2003). While it is established that different plants produce different specialized metabolites, the underlying causes determining these differences remain unknown. Two major hypotheses have been proposed, as outlined below.

The “escape-and-radiate” hypothesis (ERH) predicts sequential cycles between plant and herbivore/pathogens, with plants increasing in chemical complexity over evolutionary time, and the evolution of novel traits that promote speciation are incremental and directional throughout the diversification process (Ehrlich and Raven, 1964; Berenbaum, 1983; Agrawal, 2007).

The “resource availability hypothesis” (RAH) predicts that plants will invest more in defense when the cost of tissue replacement is high (Janzen, 1974; Coley et al., 1985; Fine et al., 2006). Therefore, in harsher, nutrient poor environments, there will be a greater investment in physical and chemical defense mechanisms over evolutionary time. For example, sparsely vegetated harsh soil is associated with increase in environmental stresses from greater exposure to droughts and herbivores, and is also expected to increase investment in chemical defenses or other morphological or physiological adaptations such as crypsis, escape, mimicry, etc. (Strauss and Cacho, 2013; Cacho et al., 2015).

Contrary to the ERH, the RAH was developed using different species rather than a single species. Furthermore, there is little evidence of this process within species under natural conditions. Early experiments within plantations of *C. calisaya* in Java, outside the natural habitat (Winters et al., 1947; Loustalot and Winters, 1948), found alkaloid content to increase with increasing soil nitrogen, while low levels of soil moisture caused a decrease in alkaloids, a clear contrast to the RAH (Arechavaleta et al., 1992; Malinowski et al., 1998).

Variation within species of plant chemical defenses has been the source of extensive investigations (Andrew et al., 2007; Barton and Koricheva, 2010; Kim et al., 2011; Holeski et al., 2012; Weldegergis et al., 2015). However, these studies have been mostly carried out in the laboratory under controlled conditions, reducing the complexity of interactions and variables that exist in native natural habitats (Moore et al., 2014). Additionally, the effect of genotypic variation within species on chemical profiles has not been studied extensively (Bidart-Bouzat and Kliebenstein, 2008). When these have been performed, genotypes have often been considered populations from separate geographical locations, leaving the genetic variation among individuals untested. Whether plant genotypes vary in their chemical defenses in *Cinchona* remains untested, although genotypic variation could potentially explain the substantial variation in alkaloids found within *C. calisaya* (Townley, 1922; Holland, 1932).

In addition to the development over evolutionary time frames, chemical defenses against pathogens and herbivores may be induced over a matter of hours or even minutes in response to external stimuli such as herbivory (Boege and Marquis, 2005; Barton and Koricheva, 2010) or over longer timespans in response to changing abiotic conditions (e.g., soil properties, climate, altitude) or biotic interactions (other plants, microorganisms) (Karban and Baldwin, 2007; Moore et al., 2014). The exact role of *Cinchona* alkaloid production in *C. calisaya* is unknown, but has been shown in laboratory experiments to inhibit larval growth (Green et al., 2002). It is thus hypothesized that quinine and the other *Cinchona* alkaloids are up-regulated in the presence of insects to inhibit their potential harmful activity on the trees.

The goal of this study is to explore the importance of genetic and environmental variation (such as climate, soil properties, and geographical distance) in determining variation of plant chemical defenses under natural conditions in *C. calisaya*. We also assess the applicability of the RAH in explaining resource allocation to chemical defense within a single species.

## Materials and methods

### Collection of *Cinchona calisaya* samples in Bolivia

**Figure 3:**
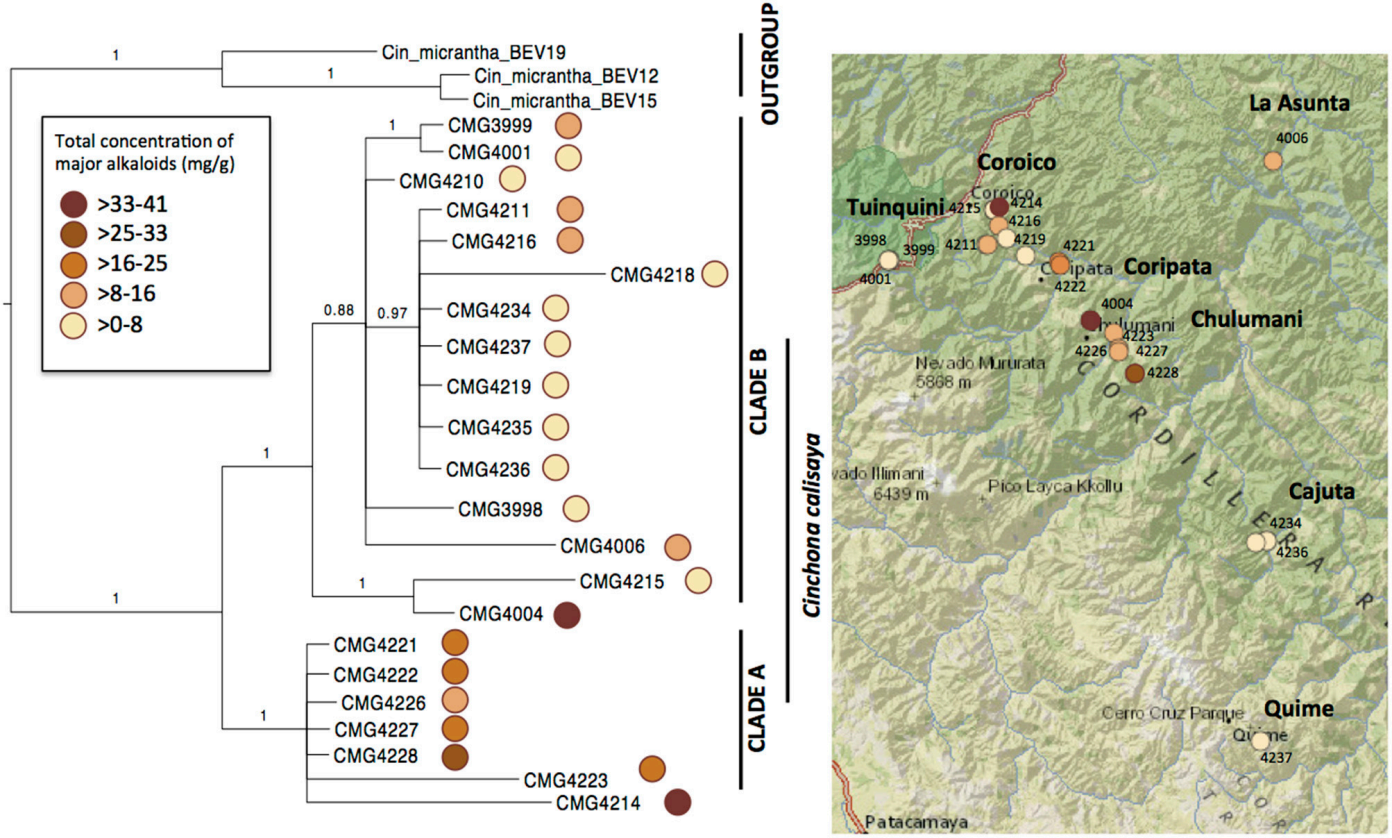
**Bayesian phylogram (50% majority-rule consensus) of the 22 specimens of ***Cinchona calisaya*** based on plastid (trnL-F, matK, rps16, and ndhF) and nuclear (ITS) sequences**. The circle coloration represents the concentration of the total *Cinchona* alkaloids found in each sample on the tree and on the map (Table 4). The reference map shows the distribution of the sampled species.

*C. calisaya* trees grow as isolated individuals or in clusters of a few trees and due to overharvesting and deforestation, *C. calisaya* trees can no longer be found at many historical sites (Ruiz, 1792; Andersson, 1998; Honigsbaum, 2001) present. Consequently the trees are increasingly hard to find. We revisited sites in the Yungas region of Bolivia, where most historical records occur (Andersson, 1998; Maldonado et al., 2015, Figure 1), and sampled *C. calisaya* trees in October 2012 and 2014 (Figure 3; Table 1).

**Table 1:** **Summarize information of Cinchona specimens sampled for this study in La Paz-Bolivia**.

**Collector**	**Code**	**Province**	**Elevation (m)**	**Latitude**	**Longitude**	**Collection date**	**DBH (cm)**	**Height (m)**
***Cinchona micrantha*** **RUIZ & PAV**.								
Escobari	BEV-12	Pelechuco	2786	−14.766350	−68.98724	19-Nov-13	9.2	11
Escobari	BEV-15	Pelechuco	2695	−14.767190	−68.98716	19-Nov-13	9.2	8
Escobari	BEV-19	Apolo	2600	−15.130694	−68.48211	17-Apr-14	9.0	5
***Cinchona calisaya*** **WEDD**.								
Maldonado	CMG-3998	Nor Yungas	1820	−16.276111	−68.06333	23-Oct-12	7.0	7
Maldonado	CMG-3999	Nor Yungas	1800	−16.280555	−67.86944	23-Oct-12	5.0	6
Maldonado	CMG-4001	Nor Yungas	1800	−16.280555	−67.86944	23-Oct-12	1.5	1
Maldonado	CMG-4004	Nor Yungas	1760	−16.380994	−67.51694	24-Oct-12	3.0	4
Maldonado	CMG-4006	Nor Yungas	747	−16.114041	−67.19970	24-Oct-12	5.0	5
Maldonado	CMG-4210	Coroico	1914	−16.254944	−67.69822	9-Oct-14	4.0	4
Maldonado	CMG-4211	Coroico	1914	−16.254361	−67.6975	9-Oct-14	8.0	6
Maldonado	CMG-4214	Coroico	1900	−16.196300	−67.68601	12-Oct-14	15.0	6
Maldonado	CMG-4215	Coroico	1900	−16.196300	−67.68601	12-Oct-14	10.0	4
Maldonado	CMG-4216	Coroico	1910	−16.224500	−67.67655	14-Oct-14	2.5	3
Maldonado	CMG-4218	Coroico	1900	−16.244027	−67.66419	14-Oct-14	2.0	3
Maldonado	CMG-4219	Coroico	1683	−16.273361	−67.62977	14-Oct-14	3.0	3
Maldonado	CMG-4221	Coroico	1348	−16.284194	−67.57266	14-Oct-14	1.5	2
Maldonado	CMG-4222	Coroico	1134	−16.288750	−67.56900	14-Oct-14	1.0	1
Maldonado	CMG-4223	Chulumani	1123	−16.402888	−67.47805	15-Oct-14	3.0	3
Maldonado	CMG-4226	Chulumani	1264	−16.428750	−67.46738	15-Oct-14	5.0	6
Maldonado	CMG-4227	Chulumani	1209	−16.433388	−67.46844	15-Oct-14	1.5	2
Maldonado	CMG-4228	Chulumani	1260	−16.472055	−67.44008	15-Oct-14	2.5	2
Maldonado	CMG-4234	Cajuata	2092	−16.750694	−67.20977	15-Oct-14	2.0	1
Maldonado	CMG-4235	Cajuata	2092	−16.750694	−67.20977	15-Oct-14	3.0	2
Maldonado	CMG-4236	Cajuata	1947	−16.753305	−67.22875	15-Oct-14	2.5	2
Maldonado	CMG-4237	Cajuata	2050	−17.084916	−67.22097	15-Oct-14	2.0	2

*DBH, Diameter at breast height*.

A total of 22 specimens were sampled for this study (Table 1). The sampled trees varied in height from 1 to 7 m with 3 m as average (Table 1) and for each individual, three samples of bark were collected at three different heights of the trunk (0.7, 1.3, and 2.0 m; Figure 2b) for chemical analysis to account for possible variation along the tree (Hodge, 1946; Nair, 2010). Trees below 2 m were only sampled at available heights.

Leaf samples were collected in silica for DNA analyses. Three soil samples were collected around each tree at 10 cm of depth (see Environmental variables section for details). The voucher specimens are deposited in the Bolivian National Herbarium (LPB) and duplicates in the Natural History Museum of Denmark, University of Copenhagen Herbarium (C).

### Detection of alkaloids in the bark

Alkaloids were extracted from all bark samples across the three heights and a test of similarity in content of alkaloids across these three heights was conducted. A few bark samples were covered in lichens, which were brushed off before the bark samples were ground to homogeneity using a custom made grinding device (Hansen et al., 2015), and 50.0 mg of the ground bark was dissolved in 1.0 mL DMSO (dimethyl sulfoxide), and ultrasonicated for 10 min. Following Holmfred et al. (in press), 4.0 mL of 70% methanol containing 0.1% formic acid was added and the mixture was ultrasonicated for 10 min. After centrifugation at 5,000 rpm for 10 min, the clear liquid phase was transferred into a 50.0 mL volumetric flask. The extraction was repeated and the liquid phase transferred into the same volumetric flask and the total contents diluted to 50.0 mL with a solution of 0.1% formic acid in demineralized water (Milli-Q). Recovery from the two extractions was 70–73 and 26–28% respectively (Holmfred et al., in press).

Whereas the official assay of the European Pharmacopoeia for Cinchona bark (Council of Europe, 2016) detects the major alkaloids as two pairs of diastereoisomers, we used an optimized extraction and HPLC method allowing separation and quantification of all four major alkaloids (Figure 2c; Holmfred et al., in press). HPLC analyses were performed with an Agilent 1200 system consisting of an on-line degasser G1379B, a binary pump G1312B, an autosampler G1367C, a column oven G1316B, a DAD detector G1315C, and a FLD detector G1321A. The column used was a KINETEX *XB-C18* (150 × 2.1 mm) with 2.6 μm particles. The gradient elution was performed using mobile phase A: 0.2 M ammonium formate buffer with pH 3.0 and water (10:90 v/v) and mobile phase B: 100% methanol at a flow of 0.2 mL min^−1^. The gradient was 18% B from 0 to 10 min, then changed to 35% B from 10 to 25 min returning to 18% B after 26 min with a total run time of 40 min. The column oven temperature was 50°C and the injection volume 3.0 μL. For UV detection: 250 and 330 nm and fluorescence detection: Excitation 330 nm and emission 420 nm was used. Quinine sulfate and cinchonine standards were obtained from Merck (Darmstadt, Germany). Quinidine and cinchonidine were both obtained from Fluka (Sigma-Aldrich, Denmark). Limit of quantification (LOQ) of the method is 5 μg/g for the four major alkaloids and repeatability of the method, expressed as the relative standard deviation (RSD) of the reference standards, was typically found to be less than 5% (Holmfred et al., in press). Total major alkaloid content was summarized and expressed in mg/g (**Table 4**).

### DNA sequencing and phylogenetic inference

To assess phylogenetic patterns of plant defenses, we produced a molecular phylogeny including 22 specimens of *C. calisaya* and three specimens of *Cinchona micrantha* Ruiz & Pav. as outgroup. Total genomic DNA was extracted from 20 mg dried leaf material using a DNeasy Plant Mini Kit (Qiagen, Denmark) with lysis of the DNA performed by incubating in a standard lysis buffer according to the manufacturer's protocol.

Four chloroplast (trnL-F, matK, rps16, and ndhF) and one nuclear (ITS) DNA regions were sequenced using the primer sets listed in Table 2. Selection of sequence regions was based on previous studies of related taxa showing useful proportions of phylogenetically informative sites within the tribe (Andersson and Antonelli, 2005; Manns and Bremer, 2010). PCR amplification was performed in a 25 μL reaction mixture containing 12.5 μL GMIX (Epicentere an Illumina Company, Madison, WI, USA), 9.5 μL H_2_O, 1 μL 20 μM of each primer, 1 μL DNA and 0.125 μL of VWR Taq DNA polymerase (VWR international) using the PCR programs described in Table 2. PCR products were purified using the QIAquick PCR Purification Kit (Qiagen, Denmark) before sequencing on an AB3130 automated sequencer (Applied Biosystems, Foster City, CA, USA). All products were sequenced in both forward and reverse directions using BigDye *v 3.1* sequencing reagents and protocols (Applied Biosystems, Foster City, CA, USA). Sequences were assembled using GENEIOUS *v R6-7* (www.biomatters.com). All sequence data were submitted to NCBI/GenBank and the accession numbers are listed in Table 3.

**Table 2:** **List of primers used in this study**.

**DNA region**	**Primer names**	**Sequence 5′–3′**	**Reference**	**Thermocycling protocol**
ITS	18S-1830F 26S-25R	5′-AACAAGGTTTCCGTAGGTGA-3′ 5′-TATGCTTAAAYTCAGCGGGT-3′	Nickrent et al., 1994	Activation of the polymerase at 95°C for 3 min followed by 35 cycles of denaturation at 95°C for 30 s, annealing at 57°C for 20 s, extension at 72°C for 45 s, and a final extension at 72°C for 7 min
				
*trnL-F*	cF fR	5′-CGAAATCGGTAGACGCTACTACG-3′ 5′-ATTTGAACTGGTGACACGAG-3′	Taberlet et al., 1991	Activation of the polymerase at 94°C for 3 min followed by 35 cycles of denaturation at 94°C for 1 min, annealing at 50°C for 1 min, extension at 72°C for 1 min, and a final extension at 72°C for 7 min
				
*rps16*	rpsF rpsR2	5′-GTGGTAGAAAGCAACGTGCGACTT-3′ 5′-TCGGGATCGAACATCAATTGCAAC-3′	Oxelman et al., 1997	Activation of the polymerase at 95°C for 3 min followed by 35 cycles of denaturation at 95°C for 30 s, annealing at 55°C for 30 s, extension at 72°C for 45 s, and a final extension at 72°C for 7 min
				
*trnK-matK-trnK*	matK1198f	5′-CTGTGTTAGATATACNAATACCCC-3′	Andersson and Antonelli, 2005	Activation of the polymerase at 94°C for 4 min followed by 35 cycles of denaturation at 94°C for 1 min, annealing at 50°C for 1 min, extension at 72°C for 2.5 min, and a final extension at 72°C for 7 min
	matk2053r	5′-TTAGCRCAAGAYAGTCGAAGTA-3′		
*ndhF*	1320f	5′GGGATTAACYGCATTTTATATGTTTCG-3′	Rydin et al., 2008	Activation of the polymerase at 95°C for 3 min followed by 35 cycles of denaturation at 95°C for 1 min, annealing at 50°C for 1 min, extension at 72°C for 1 min, and a final extension at 72°C for 7 min
*ndhF*	1000r	5′-CCTAGAGCTAGCATCATATAACCC-3′		

**Table 3:** **List of samples used for molecular analyses together with their GenBank accession numbers and voucher information**.

**Code in the paper**	**Voucher number**	**Genbank accession number**				
		**ITS**	***trnL-F***	***matK***	***rps16***	***ndhF***
***Cinchona micrantha*** **RUIZ & PAV**.						
BEV12	Escobari 12	KY676294		KY676292	KY676221	KY676238
BEV15	Escobari 15	KY676295	KY676252	KY676293	KY676222	KY676239
BEV19	Escobari 19	KY676296	KY676267	KY676269	KY676223	
***Cinchona calisaya*** **WEDD**.						
CMG3998	Maldonado 3998	KY676314	KY676254	KY676277	KY676212	KY676226
CMG3999	Maldonado 3999	KY676305	KY676268	KY676278	KY676213	KY676227
CMG4001	Maldonado 4001	KY676306	KY676266	KY676290	KY676210	KY676228
CMG4004	Maldonado 4004	KY676299	KY676255	KY676279	KY676214	KY676225
CMG4006	Maldonado 4006	KY676297	KY676256	KY676280	KY676215	KY676231
CMG4210	Maldonado 4210	KY676307	KY676257	KY676281	KY676211	
CMG4211	Maldonado 4211	KY676308	KY676258	KY676282	KY676201	KY676232
CMG4214	Maldonado 4214	KY676318	KY676245	KY676270	KY676224	KY676244
CMG4215	Maldonado 4215	KY676298	KY676265	KY676291	KY676209	KY676242
CMG4216	Maldonado 4216	KY676309	KY676259	KY676283	KY676202	
CMG4218	Maldonado 4218	KY676310	KY676253	KY676284	KY676203	KY676243
CMG4019	Maldonado 4019	KY676315	KY676260	KY676285	KY676204	KY676229
CMG4221	Maldonado 4221	KY676300	KY676247	KY676271	KY676216	KY676236
CMG4222	Maldonado 4222	KY676301	KY676248	KY676272	KY676217	KY676240
CMG4223	Maldonado 4223	KY676313	KY676246	KY676275	KY676200	
CMG4226	Maldonado 4226	KY676302	KY676249	KY676273	KY676220	
CMG4227	Maldonado 4227	KY676303	KY676250	KY676274	KY676218	KY676241
CMG4228	Maldonado 4228	KY676304	KY676251	KY676276	KY676219	KY676237
CMG4234	Maldonado 4234	KY676311	KY676261	KY676286	KY676205	KY676233
CMG4235	Maldonado 4235	KY676316	KY676262	KY676287	KY676206	KY676230
CMG4236	Maldonado 4236	KY676317	KY676263	KY676288	KY676207	KY676235
CMG4237	Maldonado 4237	KY676312	KY676264	KY676289	KY676208	KY676234

*All vouchers are deposited in the National Herbarium of Bolivia (LPZ) with duplicates at the Natural History Museum of Denmark (C)*.

Sequences were aligned using MAFFT (Multiple Alignment using Fast Fourier Transform) *v 7.017* (Katoh et al., 2002) as implemented in GENEIOUS. Using the same program, the alignment ends were trimmed manually. Data were partitioned into nuclear (ITS) and plastid (trnL-F, matK, rps16, and ndhF) regions and each region was allowed partition-specific parameters. The best suitable evolutionary model was selected according to the Akaike information criterion (AIC) and conducted independently for each partition using the program jModelTest (Guindon and Gascuel, 2003). Phylogenetic relationships were inferred using a standard Bayesian approach as implemented in MrBAYES *v 3.2.2* (Huelsenbeck and Ronquist, 2001). Analysis consisted of 3,000,000 generations, two chains, and a sampling frequency of 100 (mcmc NGEN = 3,000,000, SAMPLEFREQ = 100, NCHAINS = 2). Trace files were assessed in TRACER *v 1.5* (Rambaut and Drummond, 2007) to ensure stationarity had been obtained and to select an appropriate burn-in phase (in this case the first 10,000 sampled trees).

### Phylogenetic signal in alkaloid production

Phylogenetic signal was assessed for each of the four major alkaloids independently, as well as for cumulative total major alkaloid content using Blomberg's K (Blomberg et al., 2003), a standard index developed for continuous traits. *K* values of 0 (*K* = 0) suggest random dispersal of the trait across the tree and therefore no correspondence with phylogeny, while values greater than one (*K* > 1) suggest a correlation between the phylogeny and the evolution of the trait under Brownian motion. Statistical significance was tested by random shuffling of tips one million times using the 50%-Majority Rule Bayesian consensus phylogeny and the PHYLOSIGNAL function in the PICANTE *R* package (Kembel et al., 2010; Venables et al., 2014). A 1,000 trees were extracted from within the 95% highest posterior density region of the Bayesian trees, randomly subsampled, and Blomberg's K calculated for each phylogeny using *R*.

### Environmental variables

In the present study, “environmental parameters” refer specifically to three types of abiotic macro-environmental variables (Moore et al., 2014): Climate, soil properties, and geographical distance.

Climate variables were taken from WorldClim data (Hijmans et al., 2005). Mean annual temperature and precipitation were downloaded to 30 s resolution based on GPS coordinates of the samples, as measured in the field. Additionally, due to the large altitudinal range of the study species (200–3300 m, Figure 1), altitude and geographical coordinates were recorded by GPS (GARMIN *eTrex H*) in the field for each sample.

For the soil properties analyses, each of the three subsamples were sieved to under 2 mm before 35 g of the resultant powder was pooled together and sent for analysis by Eurofins (Denmark) for pH, humus content, and content of carbon (total), magnesium, nitrogen (total), phosphorous, and potassium. All methods are standard environmental analyses following the guidelines from the Danish Agrifish Agency under the Ministry of Environment and Food of Denmark (Sørensen and Bülow-Olsen, 1994). Humus was determined with an automated analyzer following the method of Ter Meulen as CO_2_ formed by combustion in a muffle furnace (Nabertherm, Germany) in a CO_2_ free environment (LECO Tru Mac N, Michigan). Total nitrogen was determined by LECO Tru Mac N as ammoniacal nitrogen NH_4_-N by distillation and titration following destruction of organic matter with sulfuric acid. Phosphorus was determined spectrophotometrically using a Foss, FIAstar 5000 Analyzer (Olsen's P), Potassium by flame photometry and Magnesium spectrophotometrically using (AA) ICP-OES, Thermo Scientific, iCAP 6500 Radial, by inductively coupled plasma optical emission spectrometry. Soil pH was determined using an ACCUMET *AB15* (Fischer Scientific, UK) and automated with a custom built robot.

### Multivariate analyses of explanatory parameters

The alkaloid variation was analyzed against environmental variables. Due to the large number of explanatory variables compared to dependent variables, principal component analysis was performed on climatic and soil properties (Borcard and Legendre, 2002) to reduce the number of explanatory variables. Additionally a Principal Coordinates Neighborhood Matrix (PCNM) was established from GPS coordinates for each sample. Principal components were used in subsequent multivariate analyses, with components added until over 90% of variation in each ordination was accounted for. Thus, a single component from the climatic ordination, two from the soil and two geographical, were analyzed against the concentration of the major alkaloids with PERMANOVA, using the Adonis function of the vegan package in *R* and 9,999 permutations (Oksanen et al., 2013). To test for any significant effect of sampling over different years on the alkaloid production of *C. calisaya*, sampling year was included as an explanatory factor in a PERMANOVA test to account for effects of sampling over different years. Additionally, as much of the variation of individual parameters is lost in performing ordinations used within the PERMANOVA, correlations between individual alkaloids (and total major alkaloid content), and individual climatic parameters using Spearman's rank-order correlation coefficients were used to expand upon the results found within multivariate analysis.

### Disentangling phylogenetic and environmental variation on alkaloids

Due to the presence of significant phylogenetic structure, phylogenetic generalized least squares (PGLS; Martins and Hansen, 1997) analyses were performed on the environmental parameters that significantly correlated with *C. calisaya* alkaloid content, partitioning their effects from those of phylogeny. PGLS was performed using 1,000 trees taken from the 95% posterior density region of the Bayesian trees, randomly subsampled, as with testing for phylogenetic signal with Blomberg's K.

## Results

### Variation in climatic and soil parameters

Our sampling sites covered a range in altitude with samples collected from 747 to 2,092 m above sea level (Table 4). Annual precipitation also varied greatly between sampling locations, from 558 to 1,810 mm year^−1^, and correlated negatively with altitude (*r* = −0.620, *P* < 0.001). Despite average annual temperature varying between 10.3 and 17.5°C across sites, temperature did not correlate with altitude or precipitation (*r* = −0.132, *P* = 0.269, and *r* = −0.107, *P* = 0.309).

**Table 4:** **Environmental parameters of the four major alkaloids in the bark of the ***Cinchona calisaya*** trees**.

**ID**	**Climatic parameters**			**Soil properties**							**Major *Cinchona* alkaloids (mg/g)**				
	**Temp (°C)**	**P (mm)**	**Alt (m)**	**Humus (%)**	**C (%)**	**N (%)**	**pH**	**P (mg kg^−1^)**	**K (mg kg^−1^)**	**Mg (mg kg^−1^)**	**Cinchonine**	**Cinchonidine**	**Quinidine**	**Quinine**	**Total**
3998	15.33	638	1820	14.0	85.7	0.45	4.7	0.9	13.0	9.3	4.55	0.00	0.69	1.56	6.79
3999	11.55	828	1800	16.0	83.7	0.53	5.2	0.0	10.0	7.4	0.00	0.00	3.22	5.23	8.45
4001	11.55	828	1800	16.5	84.5	0.50	4.6	0.0	7.2	2.2	0.27	0.00	0.41	0.91	1.60
4004	11.55	828	1760	6.3	93.7	0.27	4.9	0.0	9.5	17.0	2.82	0.60	6.97	25.80	36.18
4006	15.66	1810	747	9.9	90.1	0.36	5.4	1.0	25.0	58.0	1.71	0.00	5.55	3.57	10.82
4210	10.87	1180	1914	26.0	74.1	0.74	4.1	0.0	26.0	7.3	0.00	0.00	0.57	0.00	0.57
4211	10.87	1180	1914	28.0	72.4	1.01	4.0	0.0	23.0	3.3	0.43	0.00	1.80	7.83	10.06
4214	13.81	1392	1305	12.0	88.5	0.63	5.0	1.2	17.0	12.0	22.93	1.38	10.01	6.94	41.25
4215	13.81	1392	1305	5.8	94.2	0.20	4.9	0.0	6.0	5.2	5.63	0.00	1.13	0.00	6.76
4216	11.09	1210	1910	18.0	82.3	0.50	4.2	0.0	14.0	2.0	2.69	0.00	9.56	0.26	12.51
4218	10.27	1202	1900	40.0	60.5	1.21	4.1	0.0	20.0	2.7	0.00	0.00	0.14	0.16	0.30
4219	11.51	1227	1683	4.2	95.8	0.18	4.7	0.0	25.0	25.0	0.00	0.00	0.96	0.78	1.74
4221	13.84	1368	1348	6.0	94.0	0.19	5.7	0.0	20.0	38.0	2.10	2.28	0.66	17.77	22.82
4222	13.84	1368	1134	2.5	97.5	0.07	6.1	0.0	3.9	44.0	0.00	0.00	1.05	16.69	17.74
4223	14.20	1366	1123	6.1	93.9	0.20	4.4	0.0	3.9	88.0	1.01	1.62	0.52	12.69	15.86
4226	13.52	1309	1264	5.1	94.9	0.20	5.0	0.0	11.0	10.0	0.84	0.00	2.51	10.20	13.56
4227	13.87	1325	1209	2.9	97.1	0.11	4.7	0.0	7.5	7.3	1.73	2.94	0.39	11.28	16.33
4228	11.55	1282	1260	2.8	97.2	0.10	4.9	0.0	5.1	10.0	1.16	3.10	0.25	20.09	24.59
4234	15.03	1142	2092	4.7	95.3	0.29	5.6	0.4	15.0	34.0	0.00	0.00	0.97	1.70	2.67
4235	15.03	1142	2092	6.2	93.8	0.21	5.6	0.5	13.0	28.0	0.00	0.00	0.48	0.55	1.03
4236	15.46	1187	1947	2.8	97.2	0.15	5.1	0.0	6.8	3.4	0.00	0.00	0.93	0.54	1.48
4237	17.53	558	2050	3.3	96.7	0.14	5.9	0.0	7.3	12.0	0.00	0.00	0.79	0.52	1.31
Average											2.11	0.54	2.24	6.57	11.56

*Alkaloid content are averages of samples at three heights per tree (0.7, 1.3, and 2.0 m). Alkaloid content in our tested samples was not significantly different among the three different trunk heights (Figures 2b,d)*.

Soil properties varied greatly among sites, with humus content varying between 2.5 and 40.0%, C between 60.5 and 97.5%, and N between 0.1 and 1.2%. The soil was highly acidic, as typical of tropical regions, with pH ranging between 4.0 and 6.1. Low concentrations between 0.0 and 1.2 (mg kg^−1^) were found for P. Both K and Mg varied considerably, K between 3.9 and 26.0 mg kg^−1^, and Mg between 2.0 and 88.0 mg kg^−1^ among the sites (Table 4).

### Phylogenetic structure of *Cinchona calisaya* samples

The combined aligned DNA matrix comprised 3,700 base pairs (bp) derived from the five DNA regions: ITS (543 bp); trnL-F (839 bp); rps16 (746 bp); matK (764); and ndhF (808 bp). The topology derived from Bayesian analyses is shown in Figure 3 with Bayesian posterior probabilities indicated above the branches. We obtained two major clades of *C. calisaya* (A and B). Clade A (PP = 1.00) includes trees with high alkaloid content (13–41 mg g^−1^ cumulative of four major alkaloids) restricted to 1,100–1,350 m altitude between Chulumani and Coripata in Bolivia, except for CMG4214, which was collected near Coroico (Figure 3; Table 4). Clade B (PP = 1.00) primarily includes trees with low alkaloid content, but with a few exceptions. Clade B is a higher altitude clade ranging from about 1,900 to 2,300 m altitude (except CMG4006 from La Asunta at 747 m) and has a wider geographical range from Coroico and La Asunta in the North to Cajuta and Quime in the South. Clade B is further resolved into a subclade (PP = 1.00) consisting of CMG4004 and CMG4215 and a main clade (PP = 0.88) with the remainder of the samples.

### Variation in composition and quantity of major antimalarial alkaloids

Alkaloid content in our tested samples was not significantly different among the three different trunk heights (Figure 2d) (Kruskal-Wallis test, quinine *P* = 0.40, quinidine *P* = 0.46, cinchonine *P* = 0.32, and cinchonidine *P* = 0.24) and consequently the average of the three values for each of the four major alkaloids was used in our subsequent analyses.

Total major alkaloid content varied significantly (Wilcoxon test = 99, *P* < 0.001) between the high alkaloid containing clade A (ranging from 13.6 to 41.3 mg g^−1^, with a mean of 21.7 mg g^−1^), and the lower alkaloid containing clade B (ranging from 0.3 to 12.5 mg g^−1^, with a mean of 4.7 mg g^−1^). Within clade B, one sample CMG4004 had a higher content that was in the range of samples from clade A, with a total major alkaloid content of 36.2 mg g^−1^. Total major alkaloid variation across all samples varied from 0.3 mg g^−1^ (CMG4218, <0.1% of bark) to 41.3 mg g^−1^ (CMG4214; 4.1%) with a mean of 11.6 mg g^−1^ (1.2%), which is in the lower range of what has previously been reported in materials of natural origin (Nair, 2010).

**Figure 4:**
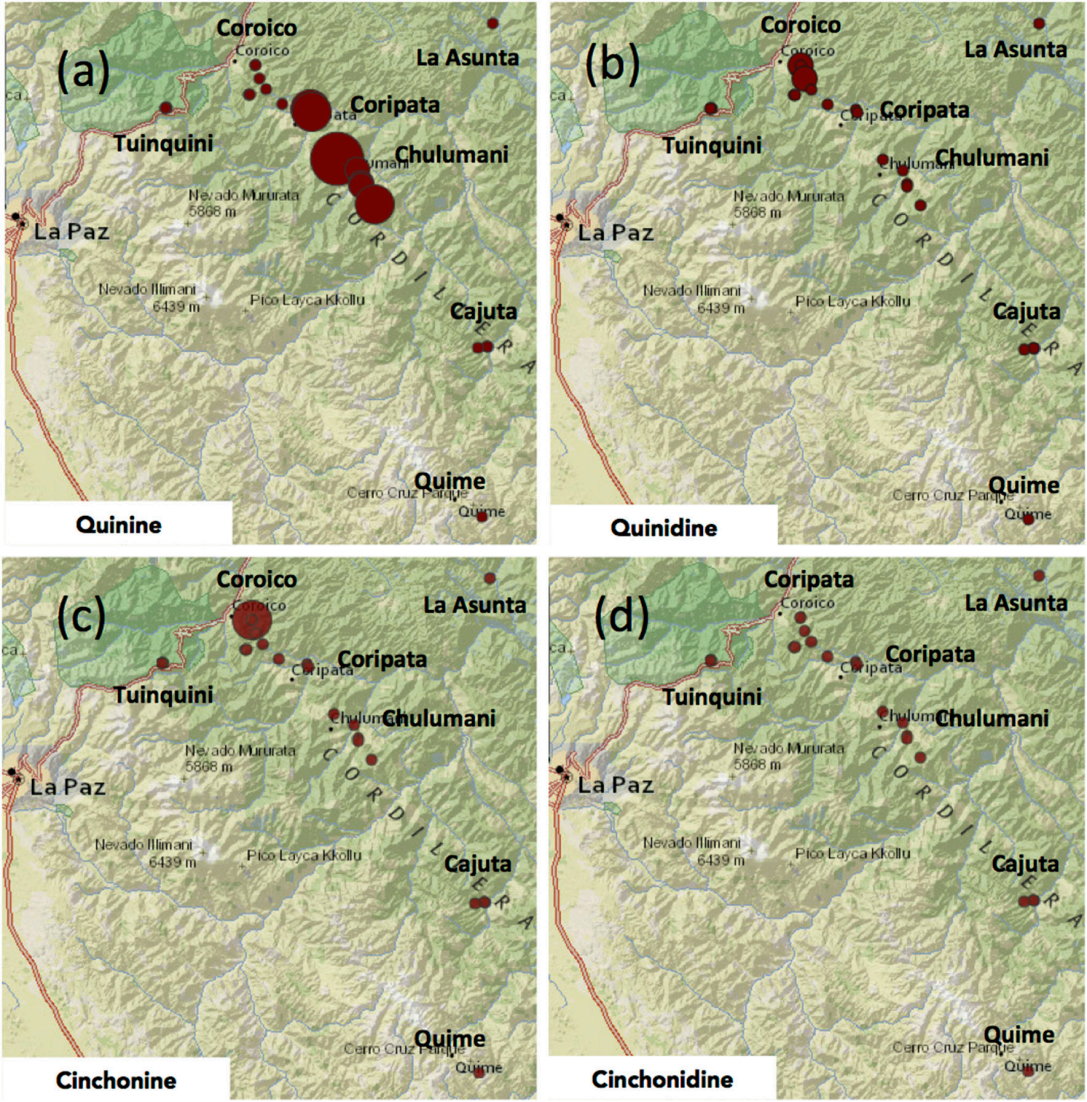
**Maps showing the relative concentration of each of the four major ***Cinchona*** alkaloids in each of the 22 samples of ***Cinchona calisaya*** across the distribution range of the included samples**. **(a)** Quinine, **(b)** quinidine, **(c)** cinchonine, **(d)** cinchonidine. The size of the colored circles corresponds to the relative concentration of the alkaloid in each sample in each panel. The absolute concentration of the four major alkaloids is listed in Table 4.

Quinine was the most abundant alkaloid with a mean of 6.6 mg g^−1^, ranging from 0.2 to 25.8 mg g^−1^ (<0.1–2.6%). While there were only two samples without any detectable levels of quinine, almost 50% of all samples tested had less than 5.0 mg g^−1^ of quinine (Table 4; Figure 4a).

Quinidine was the second most abundant of the alkaloids, accounting for an average of 2.3 mg g^−1^ (0.2%) across samples. Quinidine was present in all samples and highly variable, although less so than quinine, ranging between 0.1 and 10.0 mg g^−1^ (Figure 4b). Cinchonine accounted for an average of 2.2 mg g^−1^ (<0.2%). Like quinine, its content varied greatly, with nine trees having no content at all and half of all sampled trees having less than 2.0 mg g^−1^ (Figure 4c), with one further sample (CMG4214) having exceptionally high content of cinchonine (22.9 mg g^−1^). Cinchonidine was the least abundant of the major alkaloids, comprising an average of just 0.5 mg g^−1^ (<<0.1% of bark). Only 7 of the 22 trees sampled contained any detectable level of cinchonidine, ranging between 0.6 and 3.1 mg g^−1^ (Table 4; Figure 4d).

Spearman's rank-correlation coefficients were calculated to investigate the relationship between the content of the individual alkaloids. Cinchonidine and quinine were highly autocorrelated (*R*^2^ = 0.69, *P* < 0.001), while cinchonine and cinchonidine also correlated (*R*^2^ = 0.49, *P* = 0.020). There were however no other significant correlations among the alkaloids, with quinidine not correlating with any of the other alkaloids.

### Phylogenetic signal of major alkaloids

All four major alkaloids found within *C. calisaya* showed substantial variability among samples (Table 5). A significant phylogenetic signal was found for quinine (*K* = 1.299, *P* = 0.046), cinchonidine (*K* = 1.719, *P* = 0.002), and total major alkaloid content (*K* = 0.960, *P* = 0.020), whilst there was no correlation between phylogeny and quinidine (*K* = 0.316, *P* = 0.299) or cinchonine (*K* = 0.372, *P* = 0.325) (Table 3). Given the contrast in alkaloid content visually identified between the highly supported clade A compared to the rest of the tree (clade B), *t*-tests were performed to test for significance between the two clades. Again, quinine (*t* = 3.343, *P* = 0.004), cinchonidine (*t* = −3.277, *P* = 0.017), and total major alkaloid content (*t* = −2.739, *P* = 0.021) were significantly higher within clade A, while quinidine (*t* = 0.148, *P* = 0.885) and cinchonine (*t* = −0.992, *P* = 0.356) were not.

**Table 5:** **Phylogenetic signal of alkaloids within ***Cinchona calisaya*****.

**Variable**	***K*_(median)_**	***P*_(median)_**	***K*** **95% CI**		***P*** **95% CI**	
			**Lower**	**Upper**	**Lower**	**Upper**
**Quinine**	**1.299**	**0.046**	**1.252**	**1.357**	**0.040**	**0.054**
Quinidine	0.316	0.299	0.310	0.322	0.285	0.309
**Cinchonidine**	**1.719**	**0.002**	**1.671**	**1.784**	**0.002**	**0.003**
Cinchonine	0.372	0.325	0.365	0.380	0.315	0.337
**Total**	**0.960**	**0.020**	**0.930**	**0.992**	**0.018**	**0.023**

*K and P-values were calculated from 1,000 trees randomly subsampled from the 95% credibility set using a custom script and the phylosignal function within the package Picante of R. Median and 95% confidence intervals (CIs) are recorded for each and significant correlations are highlighted in bold*.

### Multivariate analyses of explanatory parameters

Climatic PC1 was shown to account for 20.2% of the variation within alkaloid production while geographical distance (as PCNM1) accounted for 9.7% of the variation, with a cumulative 29.9% of alkaloid variation accounted for within the analysis (Table 6). The second geographical component (PCNM2) and soil properties (soil properties PC1 and PC2) and year of sampling did not significantly influence alkaloid production.

**Table 6:** **Adonis analysis correlating the four major alkaloids against principal components for environmental variables**.

**Environmental variable**		**Df**	**SumsOfSqs**	**MeanSqs**	***F*-value**	***R*^2^**	***P*-value**
Climate	**PC1**	**1**	**1.11**	**1.109**	**5.117**	**0.202**	**0.003**
Geographical distance	**PCNM1**	**1**	**0.532**	**0.532**	**2.454**	**0.097**	**0.048**
	PCNM2	1	0.136	0.136	0.627	0.025	0.663
Soil properties	Soil properties PC1	1	0.075	0.075	0.344	0.014	0.868
	Soil properties PC2	1	0.245	0.245	1.131	0.045	0.316
	Year of sampling	1	0.135	0.135	0.621	0.025	0.658
	Residuals	15	3.253	0.217	0.593		
	**Total**	**21**	**5.485**			**1**	

*Bold indicates significance using Spearman's rank-order correlation (P < 0.05). PC1 is the principal component for the climatic parameters, PCNM1 and PCNM2 are principal coordinates of neighbors for geographical distances, and Soil properties PC1 and 2 were the first 2 principal components for the soil properties*.

**Figure 5:**
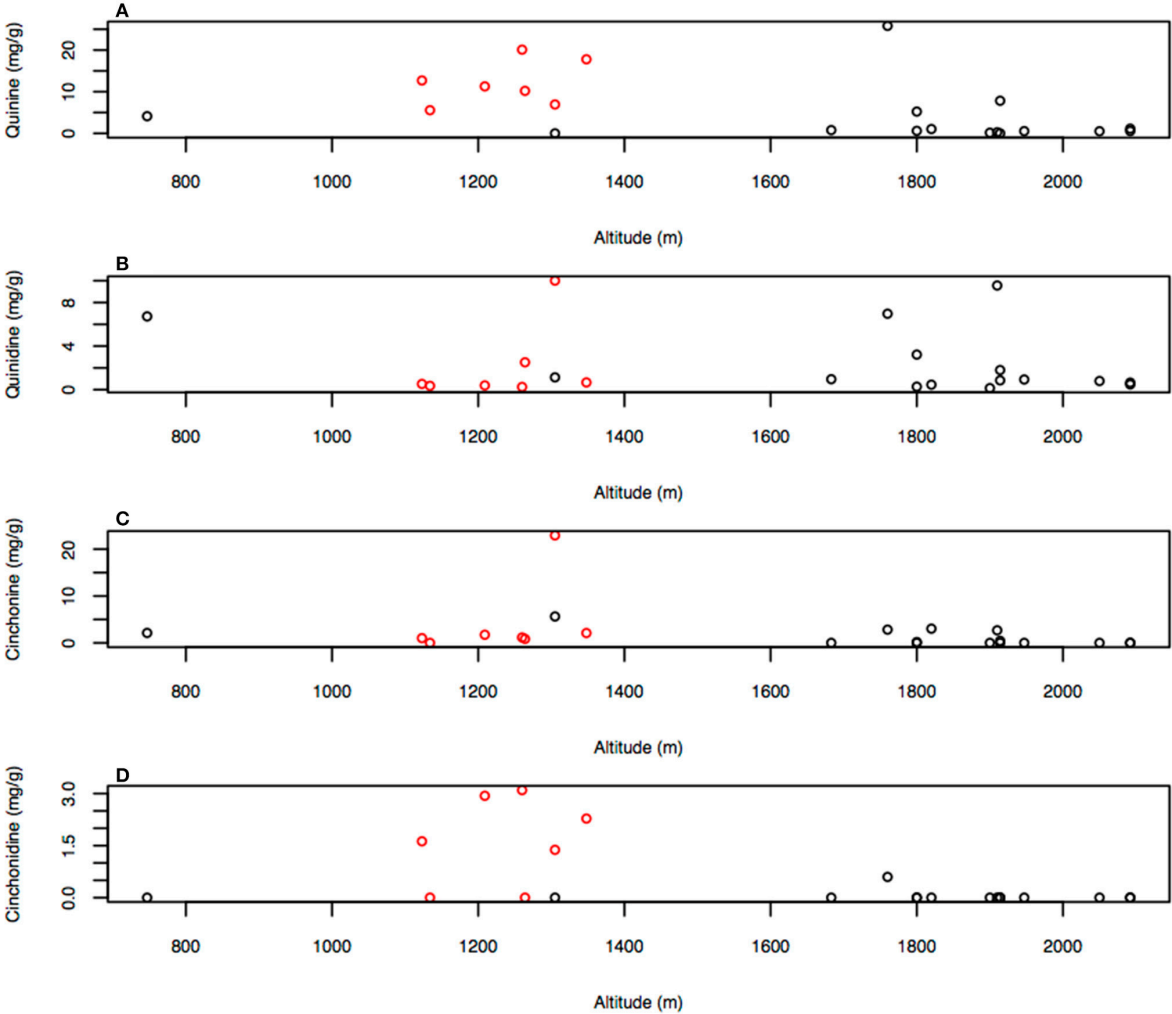
**Graph of (A)** quinine, **(B)** quinidine, **(C)** cinchonine, and **(D)** cinchonidine concentrations against altitude with markers from the high producing phylogenetic cluster in red. Correlations listed are the Pearson's rank-correlation coefficient.

The climatic PC1 significantly correlated with the alkaloid composition and closer inspection of its loadings revealed that altitude and precipitation predominantly contributed to its formation. Climatic effects on the alkaloids were investigated directly on individual alkaloid and total major alkaloid content using Spearman's rank correlation coefficient. Despite autocorrelation of altitude and precipitation, only altitude significantly correlated with any of the alkaloid concentrations, with altitude correlating with quinine (*R*_s_ = −0.548, *P* < 0.001; Figure 5A), cinchonine (*R*_s_ = −0.473, *P* = 0.026; Figure 5C), and cinchonidine (*R*_s_ = −0.507, *P* = 0.016; Figure 5D), while no correlation with quinidine was found (*R*_s_ = −0.005, *P* = 0.982; Figure 5B). Temperature did not autocorrelate with either altitude or precipitation, nor did it correlate with any of the major alkaloids.

### Disentangling phylogenetic and environmental effects on alkaloid production

Quinine, cinchonidine, and total major alkaloid content significantly correlated with phylogeny, whilst altitude also correlated with quinine content. Therefore, PGLS was performed to account for phylogenetic autocorrelation, testing for an independent altitudinal effect on quinine, cinchonidine, and total major alkaloid content. However, significance for altitude was lost on quinine (*P* = 0.236) and remained non-significant for cinchonidine (*P* = 0.851) and total major alkaloid content (*P* = 0.135).

## Discussion

Historically, quinine and quinine-like alkaloid concentrations are known to vary considerably within *C. calisaya* (Delondre and Bouchardat, 1854), however what is driving this variation is not known. This work represents one of the first attempts to simultaneously explore the relative effects of genotype and environmental variation in determining plant secondary metabolite production under natural conditions. Our results show that genotypic variation within *C. calisaya* is the primary driver of differences in alkaloid content, rather than environmental variation. A significant correlation was found between phylogeny and quinine, cinchonidine, and total major alkaloid production, whilst there was no significance for quinidine and cinchonine. Within the phylogeny, two highly supported clades were identified (clade A and clade B). Clade A was a small mostly geographically clustered group that contained all but one of the higher alkaloid producing samples, whilst clade B was comprised of a number of further subclades. Clades A and B differed significantly in alkaloid content and it is likely differences between these two drove the correlation between phylogeny and alkaloids.

Additionally, the link between phylogeny and alkaloid content may have been confounded within this work. Both the high producing sample in clade B found in Chulumani (CMG4004) and the low producing sample in clade B found in Coroico (CMG4215) were grown around settlements and were possibly cultivated plants that could have been transplanted recently from other areas, which would explain the inconsistency between geography and phylogeny.

Our results also suggested that clade A was geographically clustered, centered between Chulumani and Coripata in Bolivia. Plants are more likely to be related the closer they are geographically, therefore it is unsurprising that geographical separation correlated with alkaloid composition given the significant phylogenetic effect (Castells et al., 2005; Egydio et al., 2013). They are also more likely to share similar environmental conditions and samples in clade A were also found at lower altitudes than the other samples, ranging between 1,100 and 1,350 m. With the exception of a single sample (CMG4006), which was found at 747 m, all other samples from clade B were found above 1,700 m and produced less of the major alkaloids than the other samples in clade B. Climatic factors are thought to be amongst the most important factors determining alkaloid composition in plants (Lobo et al., 2010; Moore et al., 2014), and the negative correlation between altitude and chemical defense compounds which we found for *Cinchona* spp. has been observed before in for example the case of *Artemisia* (Fluck, 1963). However, to our knowledge a comparison between genotypic variation and environmental effects on alkaloid chemistry in the field has not been previously investigated, and in this study environmental effects were no longer significant once phylogenetic effects were taken into account. Results here therefore suggest the development of a locally adapted, highly productive genotype of *C. calisaya* in the mountain forests of western Bolivia over evolutionary rather than ecological time scales. This could therefore be compatible with the ERH (Ehrlich and Raven, 1964; Berenbaum, 1983; Agrawal, 2007), in which genotypes eventually become separate species with differing chemotypes.

Changing altitude will also affect a number of environmental properties (Del Grosso et al., 2008; Sundqvist et al., 2013), with higher rates of photosynthesis associated with lower altitudes that are generally warmer (Berry and Bjorkman, 1980; Korner and Diemer, 1987). Meanwhile, no significant effects of soil properties were observed on the four major alkaloids under natural conditions. This is in contrast to experimental manipulations, which suggest a link between alkaloid production and soil phosphorus, nitrogen and pH (Koblitz et al., 1983; Harkes et al., 1985; Arechavaleta et al., 1992; Malinowski et al., 1998), although soil properties varied under natural conditions by orders of magnitude less than the conditions established experimentally. With no significant effects of nutrient availability and potentially enhanced rates of photosynthesis of samples of clade A found in lower altitudes, our results suggest that the RAH may not apply within species despite evidence of a phylogenetic effect.

In contrast to previous studies (Nair, 2010), no significant variation was found between barks sampled at heights of 0.7, 1.3, and 2.0 m. Generally, the alkaloids are thought to be in lower content in the twigs, higher in trunk bark, and maximum in the root bark (Nair, 2010) with peak quinine content found in the collar portion (30–45 cm in length, near the base) (Hodge, 1946; Nair, 2010). However, we found limited evidence for variation in any of the four major alkaloids across the bark of the trunk.

A significant proportion of alkaloid variation remained unexplained, particularly within quinidine and cinchonine. Although temporal and biotic conditions were not studied in this work, future studies may greatly benefit from this, with sampling under natural conditions assisting in discovering novel mechanisms regulating plant secondary metabolites. For example, microbial communities in the bark have shown ability to influence production of *Cinchona* alkaloids (Koblitz et al., 1983; Maehara et al., 2010, 2011) but only *in vitro*. Furthermore, quinine and quinine like alkaloids have demonstrated activity against larvae (Green et al., 2002) *in vitro*, but no study to date has investigated the role of insect herbivory on *C. calisaya* alkaloid production using whole plants. Interestingly, insect activity and composition are known to be variable across altitudinal gradients, which peaks over the intermediate altitudes that clade A occurred, decreasing at the higher altitudes clade B was found (Rahbek, 1995, 2005; Kessler et al., 2011; Larsen et al., 2011; Pellissier et al., 2012). Further studies understanding insect biodiversity and composition could therefore be a fruitful area of research in understanding alkaloid regulation (Beccera, 2015). It should however be noted that studies under natural conditions can only imply causation and generate hypotheses, and controlled environment studies should be employed in combination with environmental sampling to maximize our understanding of plant secondary metabolite production. In conclusion, we hypothesize that phylogenetic variation is the main driver of changes in alkaloid composition. Growing plants from both clade A and B in controlled environments may help confirm this hypothesis.

## Author contributions

NR and CM conceived the ideas and supervised the research. SH, CC, and EH conducted the chemical analysis. CM conducted the fieldwork. CM assembled and analyzed the data with contributions from CB. CB designed and conducted the statistical analyses. CM wrote the manuscript with contributions from CB, NR and AA. All authors read and improved the manuscript.

## Funding

This work was supported by a grant from the Carlsberg foundation to NR, the Villum Foundation to NR and CB, and the People Program (Marie Curie Actions) of the European Unions Seventh Framework Program FP//2007/2013 under REA grant agreements (grant number PITN-GA-2013-606895)—MedPlant to NR, AA is supported by funding from the European Research Council under the European Union's Seventh Framework Program (FP/2007-2013), by the Swedish Research Council, the Swedish Foundation for Strategic Research, and through a Wallenberg Academy Fellowship.

### Conflict of interest statement

The authors declare that the research was conducted in the absence of any commercial or financial relationships that could be construed as a potential conflict of interest. The reviewer FG-F declared a shared affiliation, though no other collaboration, with several of the authors CM, CB, CC, EH, SH, NR to the handling Editor, who ensured that the process nevertheless met the standards of a fair and objective review.
